# Educational Intervention for Care Home Staff to Encourage Resident ACP

**DOI:** 10.1093/geroni/igaa057.2719

**Published:** 2020-12-16

**Authors:** Shimae Soheilipour, Gloria Gutman, Brian de Vries, Helen Kwan, Katrina Jang

**Affiliations:** 1 Simon Fraser University, Vancouver, British Columbia, Canada; 2 San Francisco State University, San Francisco, California, United States; 3 Gerontology Research Centre, Vancouver, British Columbia, Canada

## Abstract

Twenty-five Multi-Ethnic (ME) and 21 Exclusively Chinese (EC) care home staff participated in an education intervention focused on helping staff assist residents/families with goals of care conversations and other aspects of ACP in a culturally sensitive way. Surveys were completed at baseline and 6-8 week follow-up. Staff reported that the education session increased their ACP knowledge and provided strategies to help them initiate relevant ACP conversations. Registered staff (nurses, social worker, physician) demonstrated significantly higher levels of confidence to participate in and assist residents/families with goals of care conversations after compared to before training.

